# The impact of attitude toward peer interaction on middle school students' problem-solving self-efficacy during the COVID-19 pandemic

**DOI:** 10.3389/fpsyg.2022.978144

**Published:** 2022-08-25

**Authors:** Xin An, Jon-Chao Hong, Yushun Li, Ying Zhou

**Affiliations:** ^1^School of Educational Technology, Beijing Normal University, Beijing, China; ^2^Department of Industrial Education, Institute for Research Excellence in Learning Science, National Taiwan Normal University, Taipei, Taiwan

**Keywords:** attitude, peer interaction, motivation, critical thinking, problem-solving, middle school, online learning, COVID-19

## Abstract

The outbreak of the COVID-19 epidemic has promoted the popularity of online learning, but has also exposed some problems, such as a lack of interaction, resulting in loneliness. Against this background, students' attitudes toward peer interaction may have become even more important. In order to explore the impact of attitude toward peer interaction on students' mindset including online learning motivation and critical thinking practice that could affect their problem-solving self-efficacy during the COVID-19 pandemic, we developed and administered a questionnaire, receiving 1,596 valid responses. The reliability and validity of the questionnaire were re-tested, and structural equation modeling was applied. It was found that attitude toward peer interaction could positively predict middle school students' online learning motivation and critical thinking. Learning motivation and critical thinking also positively supported problem-solving self-efficacy. It is expected that the results of this study can be a reference for teachers to adopt student-centered online learning in problem solving courses.

## Introduction

The outbreak of COVID-19 has led to the widespread practice of online learning in schools (Zhao et al., 2021). Teachers and students in middle schools continue to integrate online learning into classrooms, which is further promoting the process of online and offline blended learning development (Lee et al., 2021). A conceptual ecology of learning is necessary to embrace a series of learning environment “across boundaries traditionally separating institutions of education, popular culture, home, and community” (Kumpulainen and Mikkola, 2014, p. 51). However, the coevolution of people and their environments is an ongoing process (Hilty and Aebischer, 2015). That is, online learning is intertwined with sociocultural environments (Allen et al., 2015), and when people are not able to meet physically during the COVID-19 pandemic, they need more social interactions (Kalmar et al., 2022). Peer interaction has been proved to benefit learning progression and contribute to deep learning (Chadha, 2019). Online peer interaction embedded in learning design is useful for promoting students' learning outcomes, but previous research has mainly focused on the higher education group (Lin et al., 2017; Martin et al., 2020). While online learning during the COVID-19 pandemic could have been a new experience for middle school students to interact with peers and their teachers (Clark et al., 2021), attention to K-12 education in the literature is rare, and online learning had hardly been adopted by Chinese middle schools before the COVID-19 pandemic. As the pandemic came suddenly, most teachers in Chinese K-12 schools only conducted online one-way live-streamed lectures, and did not pay attention to interactive activities, leading to surface learning and polarization of students (Yu and Wang, 2020). Thus, the present study aimed to explore the role of peer interaction in online learning based on Chinese K-12 students.

In fact, the pandemic promoted the universalization and ubiquity of online learning; thus, there is a need for more and deeper attention to online learning outcomes (Ngo and Ngadiman, 2021). Learning outcomes consist of affective outcomes, cognitive outcomes, and skill-based outcomes (Kraiger et al., 1993), of which motivation (as an affective outcome), critical thinking (as a cognitive outcome) and problem solving (as a skill-based outcome) have been the factors of most concern in the online learning research (Zhou et al., 2021). These learning outcomes are important for not only higher education students, but also K-12 students. To explore the role of peer interaction in online learning for K-12 students during the pandemic, it is necessary to study the relationship among peer interaction, motivation, critical thinking, and problem solving.

Attitude is defined as a favorable or unfavorable evaluative reaction toward something or someone, exhibited in one's beliefs, motivation, or intended behavior (Ajzen, 2005). Attitudes provide meaningful approaches to seek some degree of order, clarity, and stability in our personal motivation of reference (Harmon-Jones and Harmon-Jones, 2021). As the measurement of the real interactive behavior is difficult, students' attitude toward peer interaction is used to explore the role of peer interaction in online learning. Attitudes include affective and cognitive components to predict motivation and behavior (Ajzen, 2005). In contrast, mindsets consist of a collection of attitude judgments and cognitive processes and procedures to facilitate problem solving and completion of a particular task (Gollwitzer et al., 1990). Moreover, mindsets drive cognitive processing, and capture the critical thinking that is an important behavioral outcome judgement (Nolder and Kadous, 2018). Students' attitude toward peer interaction could have an impact on their mindsets (Zulkifli et al., 2020; Thanasi-Boce, 2021), leading to different learning performance (Kwon et al., 2019). To date, few researchers have investigated online learner profiles based on the mindset shared among students (Zamecnik et al., 2022). Online learning profiles, problem solving, critical thinking, teamwork, and motivation are different in different areas and backgrounds (Lawter and Garnjost, 2021). In particular, the profiles of middle school students who were born and grew up in the digital age, and so are known as “digital natives” (Becker and Birdi, 2018) have not been studied. In line with this, the correlates between middle school students' attitude toward peer interaction, motivation, critical thinking, and problem solving were explored in this study.

## Theoretical background

### Attitude toward peer interaction in online learning

Peer interaction is usually considered to be at the heart of the development of constructivist learning theory research (Tenenbaum et al., 2020). In peer interaction, students can question others as free and active participants in social discourse, argument, and learning (Castellaro and Roselli, 2015). The comparison of perspective will produce social cognitive conflict, and then generate consensus through interaction (Tenenbaum et al., 2020). It has been shown that peer interaction can benefit learning progression and make students learn more deeply (Chadha, 2019). Learner-learner interaction refers to the two-way communication among learners, such as exchanging ideas with classmates, discussing with each other, and getting feedback from other learners (Wang et al., 2022). Moreover, middle school students had hardly ever experienced complete online learning before the COVID-19 pandemic. When the pandemic suddenly broke out, some students could have had difficulty adapting to online learning, leading to learning anxiety, loneliness, and even depression (An et al., 2020; Perkins et al., 2021; Ying et al., 2021). Peer interaction might be the key to solving these affective and psychological problems (Yao and Zheng, 2017; An et al., 2020). Especially, social and peer influence is of great importance for adolescents (Tsai et al., 2015), so peer interaction could be an extremely important factor for middle school students' online learning. In brief, peer interaction is beneficial for students' development of knowledge and ability (Chadha, 2019), and could also help improve students' attitudes toward online learning during the COVID-19 pandemic (Ala et al., 2021; Chu et al., 2021; Ngo and Ngadiman, 2021). However, it is difficult to measure the real peer interactive behaviors of students, as the peer interactions occur naturally in social media, but are not limited to a specific learning platform. Students' attitude could show their preference for a certain behavior (Ajzen, 2005). Thus, students' attitude toward peer interaction in middle high school was explored in this study.

### Motivation as emotional mindset

The concept of mindsets is based on Dweck (1999) framework in which it was proposed that mindsets determine one's goals, motivation, and beliefs about effort. As a result, mindsets can organize associated constructs into a coherent motivational framework or “meaning system” (Yu and McLellan, 2020). An important motivational factor that might influence individuals' willingness to engage with a task is their emotional mindset (Wols et al., 2020). Students' motivation plays a fundamental role in academic achievement. In fact, Yu and McLellan (2020) applied person-centered motivation to study some key elements of the mindset-based meaning system. Although there are different ways to induce students' growth mindset, most of the studies endorsed that the growth mindset can indeed modify the learning processes (Kania et al., 2017). Learning motivation is an important factor leading to success in online learning, especially for K-12 students (Zuo et al., 2021). The COVID-19 pandemic has brought about great changes to students' learning, such as the disruption of social networks and teachers being less focused on the individual. Students in middle school have lost their traditional learning motivation sources, but have attained new motivation sources (Uka and Uka, 2020). Learning motivation can improve academic outcomes by catering to learners' needs in online learning platforms (Baker et al., 2016). Although it has been studied in much of the online learning research (Zhou et al., 2021), little research has covered learning motivation in emotional mindset under the threat of COVID-19; thus, in this research, learning motivation was studied as an emotional mindset of online learning.

### Critical thinking as cognitive mindset

Critical thinking is a kind of ability acquisition of online learning (Zhou et al., 2021), which usually refers to skills of reasoning, evaluation, analysis, judgment, conceptualization, understanding, and reflection (Guiller et al., 2008; van Laar et al., 2017). Individuals with a deliberative mindset are also more likely than those with an implemental cognitive mindset to take longer to reach a judgment (Henderson et al., 2008), indicating their openness to information and suspension of judgment. The deliberative mindset captures the mechanics behind a “questioning mind,” a “critical evaluation of evidence,” and the responsibility to “be alert” to evidence (Nolder and Kadous, 2018). In this research, critical thinking was studied as a mindset to question and to be alert for knowledge acquisition during online learning.

The Internet provides a good way for students to develop their critical thinking, as students could gain much information to help them think critically. Critical thinking is thought to be one of the most important outcomes of online learning, and previous researchers have tried to design online courses and tools to promote students' development of their critical thinking (Goodsett, 2020; Varenina et al., 2021). This study focused on self-reporting by middle school students to evaluate their cognitive mindset related to online learning.

### Problem-solving self-efficacy

Problem solving is an important ability acquisition of online learning (Zhou et al., 2021), and refers to the skills of using information and communication technology to cognitively process and understand a problem situation in combination with the active use of knowledge to find a solution to a problem (van Laar et al., 2017). Problem solving involves different skills, such as finding the nature of the problem, choosing problem-solving steps and strategies, selecting appropriate information, allocating appropriate sources, and monitoring the problem-solving process (Sternberg, 1988). Problem solving is a key competency in online learning (Aslan, 2021), and the Internet provides convenient support for learners to solve problems (Jordan and McDaniel, 2014). For learners, problem solving is the key to learning success in a future-oriented society (OECD, 2017). As little research has considered how students' peer interaction supports their problem solving, the present study would explore the relationship between peer interaction and problem solving.

In studies using self-reported scales, self-efficacy of performance or ability has been used widely in problem solving (Calaguas and Consunji, 2022). Self-Efficacy refers to individuals' beliefs about their abilities to perform expected behaviors (Bandura, 1994). According to Bandura (1997), the problem-solving self-efficacy could be defined as the students' perceptions of their problem-solving success. In previous researches, problem-solving self-efficacy (PSSE) have been used widely to represented learners' perceptions of their problem-solving abilities (Bandura, 2006; Kyung-hee, 2016; Salazar and Hayward, 2018). In this research, student' PSSE would be measured to reflect the perception of problem-solving process.

## Hypotheses

Previous studies found that online learning is useful for students, particularly in terms of learning outcomes during the COVID-19 pandemic (Jung and An, 2021; Choi-Sung, 2022). On the contrary, Hong et al. (2021a, 2022) found learning ineffectiveness through online learning, particularly in practical skill development (Hong et al., 2021b). Koehler et al. (2022) found that while students collaboratively interacted in the problem-solving process, individuals with a strong problem-solving presence valued peer interaction as an important part of the learning process, were willing to invest time engaging in the discussion, and maintained a consistent presence. Peer interaction could help to improve the motivation of students in online learning. For example, Yang and Chang (2012) found that peer interaction *via* blogs could improve students' learning motivation. Researchers found that for students in higher education, online peer interaction could improve their level of motivation during the COVID-19 pandemic (Thanasi-Boce, 2021; Kang and Zhang, 2022), but there has been a lack of focus on K-12. From this, we could speculate that for middle school students, the attitude toward online peer interaction during the epidemic could positively affect their online learning motivation. Hence, the following hypothesis was formulated in this study:

H1: The attitude toward online peer interaction could positively predict the online learning motivation of middle school students.

The mindset reflects the idea that individuals' cognitive processing determines both the content and strength of their resulting attitudes (Yu and McLellan, 2020). Critical thinking was thought to need a cognitive process, referring to the exchange and discussion of ideas with peers involved in the collaborative process of knowledge construction (Kuhn, 1991). Therefore, we could speculate that peer interaction could positively help to develop students' critical thinking. In fact, this has been proved for primary school students (Chou et al., 2015) and for undergraduate students (Oh et al., 2018; Zulkifli et al., 2020). What is more, previous researchers found that online peer discussion helped to develop university students' critical thinking better than face-to face discussion, as they would provide more well thought-out and reasonable evidence (Guiller et al., 2008). We could speculate that this might also be applicable to junior middle school students. Hence, the hypothesis formulated in this study was as follows:

H2: Attitude toward online peer interaction could positively predict the critical thinking of middle school students.

Students collaborate in groups to solve a problem, and analyze the formation of the problem to identify facts about the problem situation so that they can establish their representation of the problem and have a deeper understanding of the causes of problems. Then, they propose possible solutions, where they evaluate the gap between the current state and the desired state and address the solutions to the problem (Wu and Nian, 2021). These processes sometimes provide both an autonomy-controlling and an autonomy-supportive need to learn knowledge that may be maintained and enhanced by students' motivation (Wu et al., 2020). Moreover, individuals with a fixed emotion mindset believe that emotions are not changeable and cannot be controlled. Individuals with a growth emotion mindset believe that emotions are malleable and can be changed with effort and experience (Wols et al., 2020). For example, learning motivation is one of the major predictors of problem solving that is key in the nursing training field (Yardimci et al., 2017). To understand the correlates between middle school students' learning motivation and PSSE in online learning, the following hypothesis was proposed:

H3: Learning motivation could positively predict the PSSE of middle school students.

A problem is generally viewed as a discrepancy between desired goals and an existing state (Chi and Glaser, 1985), and problem solving is the process of taking actions to resolve this discrepancy (Shermerhorn, 2013). Researchers have suggested specific frameworks to capture effective problem solving; together, all frameworks provide insight into the critical reasoning processes learners engage in as they resolve ill-structured problems (Tawfik et al., 2020). While variation exists across focus and articulated problem-solving in online learning phases, these frameworks involve two main areas: problem finding with critical thinking (e.g., articulating a problem, constraints, clarifying diverse perspectives) and generating solutions with critical thinking (e.g., suggesting and evaluating solutions addressing identified problems) (Koehler et al., 2022). Researchers found that critical thinking had positive effects on problem solving (Kanbay and Okanli, 2017). Thus, the following hypothesis was proposed:

H4: Critical thinking could positively predict the PSSE of middle school students.

Peer interaction has an influence on problem solving because students rely on supportive social responses to enact most of their strategies to solve problems (Jordan and McDaniel, 2014). Students need to clarify or reorganize their views and plans when solving problems in peer groups, so it could be considered that the social interaction in groups is an important aspect of production power (Jordan and McDaniel, 2014). In online learning, peer interaction is also important for problem solving, but the outcome depends on the peers' competency levels and their motivation (Kwon et al., 2019). Cheng and Chau (2016) revealed that peer interaction in online learning was not at the desired level, and there was a need for studies to promote attitude toward peer interaction in online learning to enhance problem solving. However, peer interaction is one of the limitations of distance education which is widely used throughout the pandemic (Aslan, 2021). To understand how attitude toward peer interaction is related to online problem solving, in this research, the following hypothesis was proposed:

H5: For middle school students, attitude toward online peer interaction could positively predict PSSE mediated by learning motivation and critical thinking.

**Figure 1:**
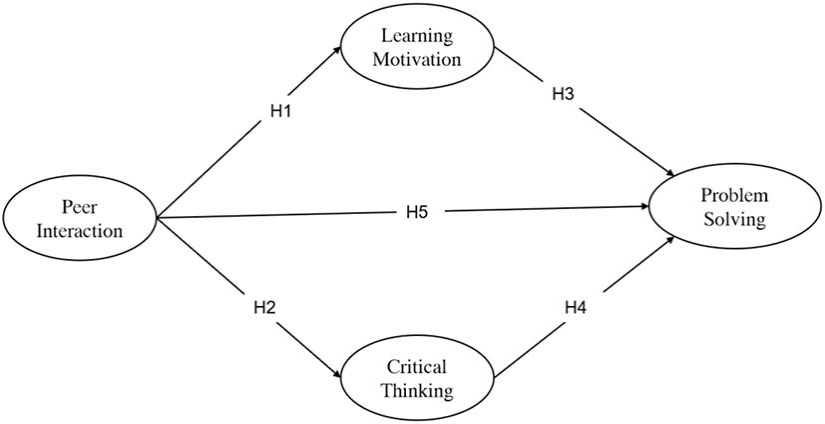
The hypothesis model.

The current study adopted a person-centered approach to examine the ways in which mindsets and associated attitude toward peer interaction constructs cohered with emotional motivation and functioned together with critical thinking as a meaningful system related to PSSE. Specifically, drawing on attitude theory and mindset theory, this study addressed the research model as explained in Figure 1.

## Method

### Participants

From July 15 to July 21, 2020, questionnaires were distributed to students in eight middle schools for the prior study, and a total of 352 sample data were collected for the EFA of the scale. The prior study samples were mainly from Beijing, Liaoning, Shandong, and Henan provinces. Invalid samples were deleted according to polygraph items, and a total of 301 valid samples were retained of which 155 (51.5%) were from boys and 146 (48.5%) from girls. There were 90 (29.9%) seventh graders, 164 (54.5%) eighth graders, and 47 (15.6%) ninth graders.

From October 20 to November 1, 2020, questionnaires were randomly distributed to junior high school students in 34 provinces or districts of China. A total of 61,419 responses were received, of which 25,805 were retained after polygraph screening. We randomly selected a similar number of different types of people in the large sample to approximate stratified sampling. A total of 1,596 valid samples were randomly selected. The demographic information of the samples is shown in Table 1. There were 803 (50.3%) boys and 793 (49.7%) girls. The proportion of students from different districts was similar, as well as that of different grades.

**Table 1:** Demographic information of participants (*N* = 1,596).

**Demographic profile**	**Classification**	**Number**	**Percent (%)**
Gender	Male	803	50.3%
	Female	793	49.7%
District	City	560	35.1%
	Town	524	32.8%
	Rural	512	32.1%
Year	Junior 1	541	33.9%
	Junior 2	538	33.7%
	Junior 3	517	32.4%

### Instrument

In this study, the questionnaire items were adapted from the relevant literature. The original measurement of peer interaction was adapted from six items of Active and Collaborative Learning in the National Survey of Student Engagement (Kuh, 2001), the measurement of learning motivation was adapted from five items of Deep Motivation in R-SPQ-2F of Biggs et al. (2001), the measurement of critical thinking was adapted from five items of the California Critical Thinking Disposition Inventory (Facione, 1990), and the measurement of PSSE was adapted from five items of Han (2020) combined with Bandura (2006) Self-Efficacy scale.

Content validity was then examined by two educational technology experts and three middle school students. Some items with unclear meaning were revised. The questionnaire used a 5-point Likert scale, with options “*strongly disagree*,” “*disagree*,” “*neutral*,” “*agree*,” and “*strongly agree*.” The higher the score, the higher the degree of agreement. After data collection, we tested the reliability and validity of the questionnaire items and constructs for subsequent structural equation modeling. The remaining items are listed in the Appendix.

### Data analysis

To further test the content validity of the scale, nine experts were invited to judge and score the relevance between each item of the scale and the construct it belongs to. A 5-point scale was used in this study, with options “*uncorrelated*,” “*weakly correlated*,” “*moderately correlated*,” “*strongly correlated*,” and “*very correlated*.” For each item, the proportion of experts who agreed that this item was strongly related to the construct (score 4 or 5) to the total number is called the Item Content Validity Index (I-CVI); it needs to reach 0.78 (Lynn, 1986).

Before data analyses were performed, normality was tested. All the measured items had appropriate skewness (ranging from −0.893 to −0.295) and kurtosis (ranging from −0.216 to 0.954), smaller than the requisite maximum values of |1| and |2|, respectively, indicating that the data of all items were close to the normal distribution (Noar, 2003).

Data analysis consisted of four stages: Exploratory Factor Analysis (EFA), Confirmatory Factor Analysis (CFA), reliability analysis, and Structural Equation Modeling (SEM). EFA and reliability analysis of the scale were conducted with SPSS20.0, and CFA and SEM were conducted with Mplus 8.3. In the prior study, 301 samples were used for EFA and reliability analysis to test the scale. In the formal study, a randomly selected subsample 1 (*n* = 799) was used for EFA, and subsample 2 (*n* = 797) was used for CFA and SEM. In the reliability analysis, all 1,596 samples were used. Bootstrapping was used 1,000 times in the indirect effect test in SEM.

In EFA, principal component analysis and the maximum variance rotation method were used to extract the factors, and components were extracted with eigenvalues > 1. If the explained variance of the first factor before rotation is <50%, it can be considered that there is no serious significant common method bias (Hair et al., 2014). Items with cross factor loadings or low loadings (<0.5) were deleted (Deng et al., 2017).

In CFA and SEM, the standards recommended by Hair et al. (2014) were adopted. Accordingly, indices of χ^2^/*df* (<5), Root Mean Square Error of Approximation (RMSEA) (<0.10), Standardized Root Mean Square Residual (SRMR) (< 0.05), Comparative Fit Index (CFI) (>0.90), and the Tucker-Lewis Index (TLI) (>0.90) were used to check the model fit degree. Then Average Variance Extracted (AVE) (>0.5) and Construct Reliability (CR) (>0.7) were calculated using factor loadings (λ) to check the convergent validity of the scale. The square root values of AVEs of components were compared with the correlations between components to check the discriminant validity of the scale. The correlations between all factors were tested for significance before SEM.

In the reliability analysis of the scale, the internal consistency coefficients' Cronbach's α values were calculated, where the whole scale and all constructs needed to be higher than 0.7 (Fornell and Larcker, 1981).

## Results

### Measurement model

In the content validity test stage, according to the nine experts' evaluation, four items were deleted as their I-CVI did not reach 0.78. A total of 21 items with good content validity were saved in the scale.

In the prior study, we conducted EFA using the 301 valid samples to explore the structural validity of the scale. Three items were deleted in three rounds of principal component analysis, as they have cross factor loadings (FL > 0.5 on two factors). The deleted items and retained items are shown in the Appendix. After that, the EFA result showed good validity. The internal consistency coefficient test showed good reliability of the scale. The Cronbach's α of the whole scale was 0.954, and the values of the subscales were between 0.850 and 0.945. All the construct reliabilities were higher than 0.7, indicating good reliability of the scale in the prior study.

In the formal study stage, the EFA results showed good validity of the scale. The Kaiser-Meyer-Olkin measure value was 0.917 (*p* < 0.001), indicating that it was suitable for factor analysis. The explained variance of the first factor before rotation was 43.520%, indicating no serious significant common method bias. A total of four main factors were obtained, and the total explained variance was 67.013%. The loadings of each item on the factor were between 0.580 and 0.823 (see Table 2).

**Table 2:** Means, standard deviations, factor loadings (λ), AVEs, and construct reliability.

**Items**	**λ-EFA**	**λ-CFA**	***M***	***SD***	**AVE**	**CR**	**α**
Peer interaction (15.203%)			3.515	0.817	0.518	0.810	0.821
PI1	0.800	0.660	3.473	1.002			
PI2	0.820	0.749	3.334	1.012			
PI3	0.643	0.823	3.623	0.975			
PI4	0.704	0.631	3.628	1.061			
Learning motivation (14.939%)			3.793	0.735	0.557	0.834	0.815
LM1	0.718	0.675	3.441	0.940			
LM2	0.779	0.726	3.708	0.947			
LM3	0.719	0.778	3.979	0.911			
LM4	0.768	0.801	4.044	0.876			
Critical thinking (16.243%)			3.839	0.724	0.550	0.859	0.854
CT1	0.580	0.765	3.804	0.920			
CT2	0.613	0.821	3.820	0.888			
CT3	0.801	0.736	3.85	0.907			
CT4	0.782	0.674	3.846	0.897			
CT5	0.744	0.703	3.875	0.861			
Problem solving (20.808%)			3.727	0.770	0.702	0.922	0.912
PS1	0.778	0.798	3.714	0.918			
PS2	0.823	0.876	3.759	0.885			
PS3	0.814	0.858	3.664	0.911			
PS4	0.774	0.836	3.760	0.885			
PS5	0.698	0.818	3.739	0.888			

The total explained variance is 67.013%. The explained variance of each component is marked in parentheses.

In the formal study stage, CFA was carried out to verify the structural validity of the scale. The model fit index of χ^2^ was 608.769, *df* was 129, χ^2^/*df* was 4.719 (<5), RMSEA was 0.068 (<0.08), CFI was 0.942 (>0.90), TLI was 0.931 (>0.90), and SRMR was 0.041(< 0.05), indicating that the fit for the items of the scale was acceptable. All standardized factor loadings were in a good range of 0.631–0.876. The values of AVE were all higher than 0.5, and CR was higher than 0.7, indicating good convergent validity. All the square root values of AVE of each component were higher than the correlations between it and other components (see Table 3), indicating good discriminant validity. All the correlations between every two factors were significant.

**Table 3:** Correlations between components and AVE of the components.

	**PI**	**LM**	**CT**	**PS**
PI	0.720			
LM	0.609	0.746		
CT	0.624	0.530	0.742	
PS	0.594	0.568	0.737	0.838

The diagonal values in the table are the square root values of AVE of each component. The non-diagonal absolute value is the correlation coefficient of each factor. All the correlations were significant (p < 0.001). PI, Attitude toward Peer Interaction; LM, Learning Motivation; CT, Critical Thinking; PS, PSSE.

In the formal study stage, the internal consistency coefficient test showed good reliability of the scale. The Cronbach's α of the whole scale was 0.920, and the values of the subscales are shown in Table 2. All the construct reliabilities were higher than 0.7, indicating good reliability. Means of components were all above the midpoint 3, as shown in Table 2.

### Structural model

**Figure 2:**
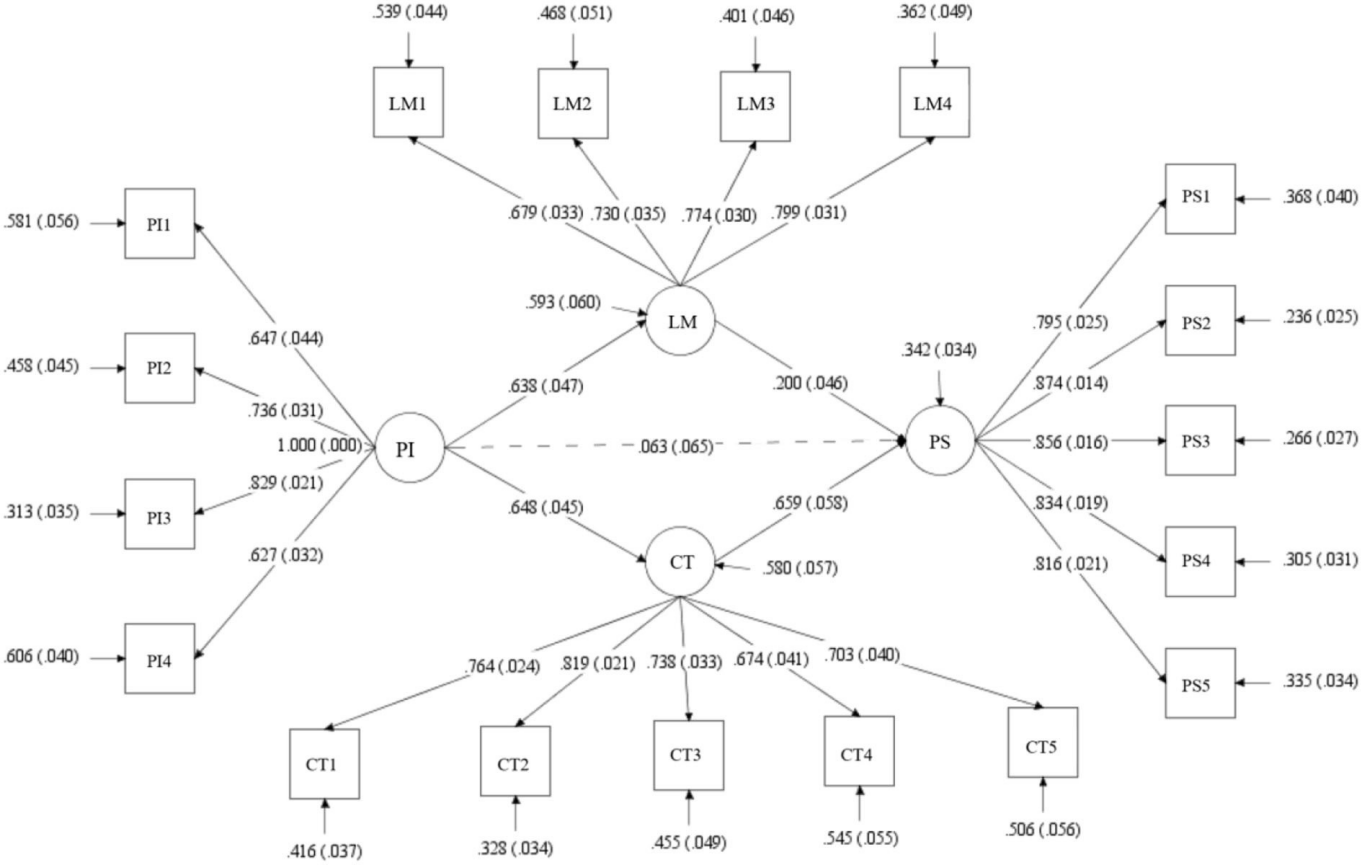
The verification of the structural model. The figure shows the SEM results of bootstrap 1,000 times. PI, Attitude toward Peer Interaction; LM, Learning Motivation; CT, Critical Thinking; PS, PSSE.

The model fit indices of the structural equation model (SEM) were good using the 797 samples for verification. The value of χ^2^ was 632.437, *df* was 130, χ^2^/*df* was 4.865 (<5), RMSEA was 0.070 (<0.08), CFI was 0.939 (>0.90), TLI was 0.929 (>0.90), and SRMR was 0.048 (< 0.05), indicating a good fit of the structural equation model. The verification of the research model is shown in Figure 2.

For middle school students, the attitude toward peer interaction could significantly positively predict their online learning motivation, of which the effect was 0.638 (*p* < 0.001), indicating that H1 was supported. Attitude toward peer interaction could significantly positively predict critical thinking, of which the effect was 0.648 (*p* < 0.001), indicating that H2 was supported. Learning motivation could positively predict PSSE, of which the effect was 0.200 (*p* < 0.001), indicating that H3 was supported. Critical thinking could positively predict PSSE, of which the effect was 0.659 (*p* < 0.001), indicating that H4 was supported.

In the path analysis, the attitude toward peer interaction did not have a significant direct effect on PSSE, of which the path effect was 0.063 (*p* = 0.331). To test the indirect effect, the bootstrap method was used. The 95% Confidence Interval (CI) was used to test whether there was an indirect effect. If the 95% CI does not include 0, an indirect effect exists (Guo et al., 2018). As shown in Table 4, the indirect effect from the attitude toward peer interaction to PSSE through learning motivation was 0.128 (*p* < 0.001), and the 95% CI did not include 0. The indirect effect from attitude toward peer interaction to PSSE through critical thinking was 0.427 (*p* < 0.001), and the 95% CI did not include 0. This indicated that attitude toward peer interaction could indirectly predict PSSE through learning motivation and critical thinking, respectively. This suggested that learning motivation and critical thinking played a full mediating role in how attitude toward peer interaction predicted PSSE. H5 was supported.

**Table 4:** Indirect effect between peer interaction and PSSE.

**Indirect path**	**β**	**SE**	**95% CI**
PI → LM → PS	0.128***	0.030	(0.058, 0.260)
PI → CT → PS	0.427***	0.049	(0.323, 0.736)
Total indirect	0.555***	0.065	(0.417, 0.900)

*** p < 0.001. PI, Attitude toward Peer Interaction; LM, Learning Motivation; CT, Critical Thinking; PS, PSSE.

The values of explanatory power (R^2^) of learning motivation, critical thinking, and PSSE were, respectively, 0.407, 0.420, and 0.658. This showed that the variables of each facet had effective explanatory power of the model, as they were above the threshold of 0.3 (Cohen, 1977).

## Discussion

A previous study indicated that participation in the peer online learning program had a positive impact on the development of communication and collaboration skills related competencies—interaction and sharing with technologies (Carvalho and Santos, 2022). Peer interaction could enhance students' engagement in online learning and increase their academic emotions (Wang et al., 2022). During the COVID-19 pandemic, it seems that online learning has greatly promoted teaching and learning reforms in the Internet environment, but problems have been exposed such as the lack of a collective atmosphere of study and social communication (Xu, 2021). Against this background, this study measured the online peer interaction of middle school students, and measured their online learning outcomes from the perspectives of psychology, knowledge, and ability. The scale used in this study had good reliability and validity. On this basis, this study explored the predictive effect of the attitude toward online peer interaction on the online learning motivation, knowledge construction, critical thinking, and PSSE of middle school students.

From the results, the attitude toward peer interaction could significantly predict the online learning motivation of junior middle school students, and the path coefficient was high at 0.645. This showed that when students in middle school had positive attitudes toward online peer interaction, they would more likely be interested in online learning and be eager to learn online. This provides support to the view of the growth emotion mindset, which posits that emotions are malleable and can be changed with effort and experience (Wols et al., 2020). The result was similar to previous studies in higher education (Yang and Chang, 2012; Thanasi-Boce, 2021), but the prediction effect of junior middle school students during the COVID-19 pandemic was much stronger. For teenagers, peer socializing is an important source of motivation for them to participate in many activities (Tsai et al., 2015). Especially during the COVID-19 pandemic, most students felt lonely and anxious during online learning (Alshammari et al., 2021). Improving students' attitude toward peer interaction could help them improve their online learning motivation.

The attitude toward online peer interaction also had a strong predictive effect on the critical thinking of middle school students, with a path coefficient of 0.670. This is consistent with previous research about university students (Guiller et al., 2008). With active attitudes toward online peer interaction, middle school students could exchange different views, question others, debate, and negotiate (Goodsett, 2020; Varenina et al., 2021). In this way, they could learn to evaluate the views of others, instead of blindly listening to authority. They would also think more from different perspectives and multiple angles, so as to develop their critical thinking. Previous research found that middle school students usually gave surface and rapid responses instead of engaging in critical thinking during online learning, even though the instructor guided them to think critically (Zhang, 2013). Improving peer interactions among students might be a useful solution to the problem.

Online learning motivation could positively predict PSSE. This is consistent with the nursing training field (Yardimci et al., 2017). The autonomy-controlling and autonomy-supportive needs to learn knowledge might arise from the problem-solving process, and be maintained and enhanced by students' motivation (Wu et al., 2020). During the COVID-19 pandemic, the level of learning motivation of middle school students might be lower than that of traditional learning, as there was a lack of peer pressure, classroom experience, and teachers' attention (Uka and Uka, 2020). To improve students' performance of problem-solving, some measures should be taken to help students maintain and enhance their learning motivation.

Critical thinking could positively predict PSSE during the COVID-19 pandemic, of which the explanation rate was the highest for predicting PSSE. When students have better critical thinking, they are more likely to find the problem and evaluate the solution to the problem reasonably (Koehler et al., 2022), which can lead to better PSSE. This is consistent with the finding of Kanbay and Okanli (2017). For middle school students, it is sometimes difficult to guide them to develop critical thinking online (Zhang, 2013), which may negatively lead to PSSE. More useful measures should be taken to improve students' critical thinking, which is the key to promoting good performance in problem solving.

Developing problem-solving ability through peer collaboration has become a focus of learning in recent years (OECD, 2017), in which motivation as emotional mindset and critical thinking as cognitive mindset should be considered. As the results of this study showed, the direct prediction of attitude toward peer interaction on the PSSE of middle school students was not significant, but the indirect effects analysis showed that learning motivation and critical thinking were two total mediating factors of the effects from attitude toward peer interaction to PSSE. This is further exploration and a complement to previous research findings (Jordan and McDaniel, 2014; Setyowidodo et al., 2020). Previous research found that peer interaction could have an impact on PSSE (Jordan and McDaniel, 2014), but did not consider the role of mindset in this process. This research explores the role of two important mindsets in this process, and showed that attitude toward peer interaction could have an effect on PSSE through learning motivation and critical thinking. This showed that the peer interaction of middle school students during the epidemic mainly played a direct role in promoting their mindsets of motivation and critical thinking, but it was difficult to directly help students solve problems. This might be due to the fact that online peer interactions among middle school students during the pandemic were mostly spontaneous interactions, with less involving online collaborative problem-solving learning activities designed and organized by teachers in China (Yu and Wang, 2020); thus, the direct prediction effect was not obvious. To explore this possibility in depth, more elaborate experimental designs are needed in the future.

Drawing on learning ecology, this study provides evidence for the importance of attitude toward peer interaction for the online learning of middle school students during the COVID-19 pandemic. The findings of this study reflect the importance of online peer interaction for middle school students during the pandemic. The results demonstrated the positive predictive effect of peer interaction on motivation and critical thinking, and reflected that the two mindsets can positively predict students' PSSE. Lacking an online collaborative problem-based activity design provided by teachers during the pandemic, online peer interaction did not directly support PSSE, but indirectly predicted it through promoting mindsets. Briefly, this study explored the relationship among attitude toward peer interaction, motivation, critical thinking, and PSSE from a new perspective in a special period. The structural model is innovative and reflects the particularity of middle school students. In the post-epidemic era, online learning has become an important learning method for junior high school students, for which peer interaction is of great significance.

## Conclusion

### Implications

This study used structural equation modeling to demonstrate the positive effect of attitude toward peer interaction on middle school students' learning outcomes during the pandemic. On the one hand, this fills the gap of previous research, that is, the lack of attention to the development of higher-order thinking in online learning in K-12 education. On the other hand, the study proposes a new model, which has a theoretically innovative role for studying peer interaction, mindsets, and PSSE in online learning.

This study provides a reference for organizing middle school students to carry out online learning. The study found that attitude toward peer interaction had a very positive predictive effect on learning mindsets and PSSE. Based on this finding, educators can guide learners to interact more with peers based on social networking sites when organizing middle school students' online learning. In practice, the scale developed in this study can be used to measure students' online learning outcomes and to adjust the design and organization of online learning in a timely manner.

### Future research suggestions

The peer interaction studied in this research was general interaction without a specific classification. In previous studies, some researchers studied peer interaction more specifically and deeply, such as whether there were task instructions for peer interaction and how many people interacted in groups (Tenenbaum et al., 2020). What is more, different kinds of interaction platforms, such as formal learning forums (MOOC forum) and social media (Facebook, Wechat) may also lead to different results. In previous research, digital natives preferred to interact with each other through the social networks that they were familiar with rather than the forum embedded in a learning platform (Butrime et al., 2015). This preference could be further studied in the future.

In addition, some researchers have studied students' competencies in more specific online learning courses, such as online nursing courses (Song et al., 2022) or person-centered online courses (Li and Tsai, 2017; Koenka, 2020). However, this study did not specify what kind of online course affects participants' cognitive and emotional factors. Future studies may explore what kinds of online courses trigger learners' emotional and cognitive mindsets in online peer interaction.

Due to the limitations of the self-report scale, the data obtained in this study may lack a certain degree of objectivity. In future research, the results of this study can be validated through learning analysis based on data collected from the learning platform.

## Data availability statement

The raw data supporting the conclusions of this article will be made available by the authors, without undue reservation.

## Ethics statement

Ethical review and approval was not required for the study on human participants in accordance with the local legislation and institutional requirements. The patients/participants provided their written informed consent to participate in this study.

## Author contributions

XA contributed to the conception of the study, performed instrument development, data analysis, and wrote the manuscript. J-CH contributed to the theoretical framework of the study and wrote the manuscript. YL contributed the instrument development, data collection, and manuscript preparation. YZ contributed to the instrument development and manuscript revision. All authors contributed to the article and approved the submitted version.

## Funding

This work described in this paper was supported by a grant from the National Social Science Fund of China (Grant Number: 21&ZD238). This work was financially supported by the Institute for Research Excellence in Learning Sciences of National Taiwan Normal University (NTNU) from the Featured Areas Research Center Program within the framework of the Higher Education Sprout Project by the Ministry of Education (MOE) in Taiwan and BNU Interdisciplinary Research Foundation for First-year Doctoral Candidates BNUXKJC2102.

## Conflict of interest

The authors declare that the research was conducted in the absence of any commercial or financial relationships that could be construed as a potential conflict of interest.

## Publisher's note

All claims expressed in this article are solely those of the authors and do not necessarily represent those of their affiliated organizations, or those of the publisher, the editors and the reviewers. Any product that may be evaluated in this article, or claim that may be made by its manufacturer, is not guaranteed or endorsed by the publisher.

## Appendix

**Table:**

**Construct**	**Items**
Attitude toward peer interaction	I like discussing problems in learning with my friends online.
	I like arguing with others on the Internet.
	I like discussing with peers to clarify the online learning content.
	I like cooperating with others to complete group tasks online.
	I have never communicated with others about learning online. (polygraph test)
	Online discussion allows me to understand the learning content more deeply. (deleted item)
	I use online tools (QQ, WeChat, email, etc.) to ask others for help when meeting problems. (deleted item)
Learning motivation	Online learning often brings me a sense of personal satisfaction.
	When learning online, I think many topics are very interesting.
	I'm eager to learn more through the Internet.
	I think there is a lot of interesting learning content on the Internet.
	I study online entirely because of parents' and teachers' requests. (polygraph test)
	I want to solve problems with the help of the Internet. (deleted item)
Critical thinking	When I have different opinions from teachers or experts, I make various inquiries.
	When I encounter problems in learning, I query and analyze from multiple angles.
	When I accept new knowledge, I will think about whether it is accurate.
	I will evaluate whether the views on the Internet are correct.
	When I have different opinions from others, I will try to change my perspective.
	I believe that the information on the Internet is all authoritative and credible. (polygraph test)
PSSE	In my study, I use the Internet to solve complex problems.
	In the face of complex problems, I can find the key to solving problems.
	In the face of complex problems, I can formulate solutions.
	When determining the solution to the problem, I always compare different solutions.
	I use all kinds of knowledge to solve the problems I meet.
	I have never solved a complex problem. (polygraph test)
